# Trait and State: Interoceptive Accuracy during Anticipation of Public Speaking in Junior Secondary Shy Students from an Eastern Province of China

**DOI:** 10.3390/ijerph18094951

**Published:** 2021-05-06

**Authors:** Jianfen Wu, Hui Li, Yunpeng Wu

**Affiliations:** 1 School of Education, Hangzhou Normal University, Hangzhou 311121, China; 2 School of Education, Macquarie University, Sydney, NSW 2109, Australia; philip.li@mq.edu.au; 3 School of Psychology, Shandong Normal University, Ji’nan 250014, China; waitu_leo@126.com

**Keywords:** interoceptive accuracy, shyness, adolescents, public speaking, heartbeat perception

## Abstract

This study investigated the interoceptive accuracy (IAc) of shy adolescents during anticipation of public speaking with a 2 × 2 factorial design. Altogether, 637 junior secondary students in an eastern province of China were sampled and screened with the Chinese version of Cheek and Buss shyness scale. The top 27% of students were considered the shy group (*n* = 30, 16 girls, *M*_age_ = 13.03, *SD* = 0.67), whereas the bottom 27% were labelled the non-shy group (*n* = 31, 16 girls, *M*_age_ = 13.16, *SD* = 0.86). The two groups of participants estimated their heart rates during specified intervals using a mental tracking paradigm in two conditions (baseline vs. anticipation), while their actual heart rates were simultaneously measured. The results indicated that: (1) the shy adolescents were more accurate in estimating their actual heart rate than non-shy adolescents; and (2) both shy and non-shy adolescents exhibit enhanced IAc in anticipation conditions when compared with baseline conditions. Implications of the higher IAc of shy adolescents and the state feature of IAc are discussed.

## 1. Introduction

### 1.1. Shyness

Shyness can be viewed as an emotional state and a personality trait [1]. As a state, it refers to arousal and heightened self-conscious emotion or feeling in response to social interaction. As a personality trait, it refers to wariness and anxiety in the face of social novelty and perceived social evaluation despite a desire to interact socially [2]. Henderson et al. summarized shyness as consisting of four components, including physiological (e.g., racing heart), cognitive (e.g., negative self-evaluation), emotional (e.g., social anxiety, fear), and behavioral (e.g., social inhibition) [3]. When facing social interaction, everyone experiences shyness, whereas those with trait shyness experience anxiety and conflict more often, and such feeling is more stable.

To enhance our understanding of shyness, we distinguish it from similar concepts. First, in contrast to behavioral inhibition that emphasizes biologically based wariness in a novel context, shyness has been defined as social wariness [4]. Second, other social withdrawal phenomena such as social disinterest or unsociability [5,6] are driven by a low approach motivation. However, a key driver for shyness is the high approach and high avoidance motivation conflict. Shy individuals wish to join the conversation, yet they often refrain from doing so because of anxiety and hesitation [2]. Third, shyness-sensitivity and regulated shyness have been investigated in Eastern, especially Chinese, cultures. In Chen and his colleagues’ studies, shyness-sensitivity was defined as wariness and anxious social reactivity (rated by peers as someone who is very shy, usually sad, and whose feelings get hurt easily) [7]. Regulated shyness refers to an acquiescent, nonassertive, and unassuming disposition when interacting with peers, and it is related to positive adjustments among Chinese children [8,9]. In this study, we applied the widely used definition of shyness similar to conflict shyness, emphasizing internal conflicted feelings and social anxiety [2]. A culturally equivalent construct allows for more valid cross-cultural comparisons. Meanwhile, focusing on negative social adjustment theoretically and empirically should contribute to our understanding of the mechanisms of shyness and benefit interventions for its negative consequences.

### 1.2. Shyness and Interoceptive Accuracy

Interoception is multifaceted and includes information processing that is conscious and non-conscious. It generally refers to one’s awareness of and ability to integrate sensory information about internal, physiological bodily states [10,11]. Garfinkel et al. proposed that interoception might have three dimensions: interoceptive accuracy (IAc; objective ability to detect interoceptive signals such as heartbeat), interoceptive sensitivity (IS; subjective beliefs about one’s IAc; assessed by questionnaires or confidence ratings), and interoceptive awareness (IAw, the meta-cognitive confidence-accuracy correspondence) [12]. This study aimed to investigate a general marker of IAc to provide insight into the ability to gauge internal bodily signals accurately.

It has been suggested that interoception plays a central role in the experience of emotion [13]. The consequences of interoception, such as increased anxiety or negative cognitions, have been found in empirical studies. False feedback of increased heart rate led to increases in self-reported anxiety and negative beliefs in individuals with social phobia [14]. Furthermore, Gerlach et al. found that an anxiety-related somatic symptom (i.e., the sound of one’s heartbeat) made public using sound speakers increased anxiety in individuals with social phobia (SP) but not in non-anxious controls [15].

IA varies across individuals and is largely considered a trait-like characteristic with high-retest reliability [16]. Prior research has identified an inverse association between IAc and depression [17], and a direct relationship between IAc and anxiety [18]. Furthermore, one study measured IAc before and during public speaking anticipation in high and low social anxiety individuals. The results suggested that the IAc of high social anxiety individuals outperform low social anxiety individuals [19]. The core features of shyness are the approach-avoidant conflict and consequent nervous and anxiety behaviors in social novelty and perceived social evaluation situations. Based on the negative consequences of sensitive interoception and empirical evidence on individual differences between high and low social anxiety groups, it can be assumed that higher IAc may play an important role in the etiology of shyness. However, whether a shy individual is more sensitive to their interoceptive cues, specifically in social situations, has not been explored.

Meanwhile, several studies have demonstrated temporary state-like changes in IA by experimental manipulation. For example, IA was improved by presenting self-relevant information such as one’s own face, self-relevant words, and a romantic partner’s face [20,21,22], and it should be noted that the effect was often observed only for those with lower IA at baseline [20,22]. In heightened self-focus situations, manipulated by mirror self-observation, the IAc of low-IAc individuals increased significantly [22]. This result indicated that an individual’s interoceptive ability might change in response to the organism’s state. Two studies investigated the effects of emotion-eliciting stimuli and/or emotional states on state IAc, yet reached inconsistent conclusions. Stevens et al. found a significant difference from baseline to the first heartbeat counting trail of anticipation phase in both high and low anxiety groups, yet failed to find significant differences from baseline to anticipation phase in either group [19]. However, in research with a typically developing population, compared with the control condition, participants exhibited increased IAc in a speech anticipation condition [23], indicating that anxiety-inducing situations (e.g., speak anticipation) or emotional states may enhance IAc.

Studies examined the modulation of IAc by more social stimulation were also incongruent. Durlik and Tsakiris showed that IA decreased after an experience of social exclusion. They suggested that social exclusion triggered a shift from predictive to reactive control, which caused attention to be oriented externally rather than internally [24]. Conversely, another study inducing the feeling of being watched by having a video camera switched on did not impact the IAc [25]. As shyness occurs in novel social interactions or social evaluation situations, which are anxiety-inducing and contain more social stimulations, a further examination of the effects of emotion-eliciting stimuli on IAc is necessary to reveal the mechanism of shyness.

### 1.3. Interoception in Shy Adolescents

Junior secondary students are adolescents undergoing a period of increased vulnerability due to reorganizational and maturational processes of the brain’s cognitive and of behavioral systems [26]. Many studies have reported the notably elevated probabilities for the onset and occurrence of mental disorders such as affective disorders and anxiety among adolescents [27,28,29,30]. Adolescence is also a critical period for the development of interoception [31]. Atypical neural activity in interoceptive networks in adolescents is described as being associated with psychopathologies, such as substance use disorders and physical health problems [32,33].

It is of great significance to examine shyness in junior secondary students. Shy students typically report more negative affect but less positive affect, experience fear of negative evaluation, tend to use self-blame attributions, and avoid social interaction [3]. Shyness was traditionally regarded as a social withdrawal case beginning in childhood [4]. It usually reached its peak level during adolescent development [34,35]. During the adolescence period, immense changes occur in one’s brain structure, hormone levels, and cognitive abilities, which lead to the rapid development of self-consciousness, enhancing the adolescent’s sensitivity to others’ evaluations [36]. Higher sensitivity to interpersonal evaluation may lead young people to exhibit a higher level of social anxiety and behavioral inhibition [37]. Against this background, this study aimed to explore the interoception of shy adolescents as a marker for vulnerability to maladjustment and to contribute to efforts at intervention.

### 1.4. Study Purpose

The main aim of this study was to compare IAc in shy and non-shy adolescents. In particular, the following questions guided this study:

1. Will the shy students exhibit higher IAc than non-shy participants?
2. Will IAc in the anticipation condition be higher than that in the baseline condition?
3. Will the shy group exhibit higher IAc gain than the non-shy group from baseline to anticipation condition?

## 2. Materials and Methods

### 2.1. Participants

Participants were recruited from 637 middle school students in a city in Shandong Province in Eastern China. The 27% of students with the highest shyness scores were enrolled as the shy group, and the 27% of students with the lowest shyness scores were enrolled as the non-shy group. From the shy and non-shy groups, 32 shy students (16 girls) and 32 non-shy students (16 girls) aged 12–14 years were randomly selected. Participants were excluded if they were currently taking medication that could influence the cardiovascular system, and none reported a history of neurological or psychiatric disorders. The trait anxiety of participants was also measured in the screening stage; individuals with severe anxiety were excluded.

**Figure 1:**
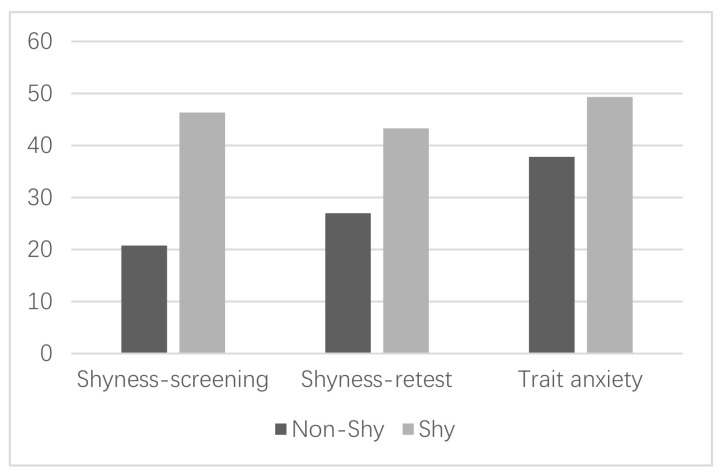
Comparison of the shy and non-shy groups.

All students volunteered to participate in this study, and written informed consent forms were obtained from all of their parents before the experiment. Three participants (one in the non-shy group, two in the shy group) did not participate due to personal reasons. The shyness level of participants was retested before the experiment. The shyness level of the shy group in both screening and retest, as well as their trait anxiety, was significantly higher than that of the non-shy group. The description of the final sample is summarized in Table 1 and Figure 1.

### 2.2. Measure

#### Self-Reported Measures

The shyness level was measured with the Chinese version of the Cheek and Buss shyness scale [38]. It includes 13 items (e.g., “I feel tense when I’m with people I don’t know well”) with a five-point Likert rating scale from 1 (strongly disagree) to 5 (strongly agree). The sum of all item scores was computed as a shyness score (score range: 13–65), with a higher score indicating a higher shyness level (Appendix A). The shyness ranges of the shy and non-shy groups were 43–51 and 18–23, respectively.

Trait anxiety was measured with the trait subscale of the Chinese Version State-Trait-Anxiety-Inventory [38], which includes 20 items with a four-point Likert rating scale from 1 (Almost never) to 4 (Almost always). The sum of all item scores was computed as a trait anxiety score (score range: 20–80) with a higher score indicating a higher anxiety level (Appendix B). The Cronbach’s α for these two measurements with the screening sample in the current study was 0.82 and 0.79, respectively.

Participants reported their anxiety mood on the visual analogue scale (VAS; 0–100) depending on how anxious, nervous and tense they felt (Appendix C).

### 2.3. Interoceptive Accuracy

IAc was measured with the mental tracking method, by asking individuals to count their heartbeats without relying on their pulse [39,40]. Participants were instructed to mentally count their heartbeats from the moment they received an audiovisual computer-generated Chinese cue “start” until they received an otherwise identical cue “stop”. Participants then orally reported the number of heartbeats they had counted. The heartbeat counting task consisted of a three-trial block: 25 s, 35 s, and 45 s, presented in random order. The single three-trial block was administered once at baseline condition and once during anticipation condition. The individual usually underestimates their heartbeat number [41]. Throughout the assessment, participants were not permitted to take their pulse. No information regarding the length of trials or feedback regarding participants’ performance was given. The task was programmed using software E-prime 2.0 (Psychology Software Tools, Sharpsburg, Washington, MA, USA).

During each trial, the actual heart rate was recorded with a Biopac MP150 integrative system (BIOPAC, Goleta, USA). Electrocardiography was collected with a wireless ECG module BN-EL30-LEAD3 from Biopac at a sampling rate of 1000 Hz from three electrodes attached to the chest. Two electrodes were active, one placed on the right collarbone and one below the left’s lowest rib. The ground electrode was placed on the right abdomen. Heart rate was derived offline using software AcqKnowledge 4.3 (BIOPAC Systems, Goleta, CA, USA) [42].

### 2.4. Procedure

The study was approved by the Institutional Review Board of Shandong Normal University. Before in-lab participation, written informed consent was obtained from the parents of all participants. Experimental sessions were scheduled between 2 pm and 5 pm and lasted about 50 min. Each participant was tested individually. After attaching the electrodes, they received the instructions for the heartbeat detection task from the experimenter. A short test trial (10 s) was administered before the formal task to familiarize participants with the task.

A 2 × 2 between-subjects factorial design was employed, with the independent variables being shyness (shy vs. non-shy) and conditions (baseline vs. experiment). The dependent variable was the participant’s perception of heartbeat. All participants completed two heartbeat detection tasks in both baseline and anticipation conditions. Further, unlike Stevens et al. [19], we counterbalanced the condition’s order to control the potential effect of task order on IAc. Participants took a 2 min break between the two experimental tasks. In the baseline condition, participants were asked to complete the detection task. In the experimental condition, the experimenter told participants that, right after completion of the detection tasks, they would be given three minutes to prepare a 3 min speech on ‘my typical day’, presented in front of two audiences and a video camera in a nearby room. To increase the task’s plausibility, participants were then given piece of scrap paper and a pencil to prepare the speech. After manipulation, each participant was asked to complete the detection task. In each condition, participants complete the VAS ratings after the detection task. For the anticipation-to-control condition order, after the detection task and VAS rating in anticipation condition, the experimenter informed the participants that the speech task was canceled due to a device error. Then participants performed the detection task in the baseline condition.

Lastly, each participant was informed that the study had come to an end, and the deception was explained to each participant. Participants were asked debriefing questions about the believability of the manipulation and the distress evoked by the manipulation. Finally, the participant received a notebook worth 10 RMB as a gift.

### 2.5. Hypotheses and Data Analysis

The widely used index of IAc is individuals’ accuracy scores on heartbeat perception tasks [39], as differences have been found between high and low social anxiety individuals [19]. As social anxiety shares some features in the somatic (e.g., blushing) and cognitive domains with shyness [43], we made hypothesis 1 for this study. Meanwhile, to examine IAc’s stability under emotional influences such as anticipatory anxiety, IAc was measured at rest (baseline), and while anticipating a speech in front of two audiences and a video camera (anticipation). As an effective method of inducing social anxiety [19,44], the anticipation of public speaking was utilized in this study. As the study on IAc with a normal population suggested that speech anticipation can enhance IAc, and higher levels of fear of negative evaluation were related to a larger increase in IAc due to anticipatory anxiety [18], we proposed hypotheses 2 and 3 for this study.

**Hypothesis** **1.** Shy participants exhibit higher IAc than non-shy participants.

**Hypothesis** **2.** IAc in the anticipation condition will be higher than that in the baseline condition.

**Hypothesis** **3.** The shy group exhibit higher IAc gain than the non-shy group from baseline to anticipation condition.

The scores of three VAS ratings were averaged into a measure of self-reported anxiety, with a higher score indicating higher general levels of anxiety. R-wave peaks were detected from an electrocardiogram trace and counted the number of R-waves that induced beats in each trial. The IAc scores were computed by means of the following formula:

IAc score = 1/3 ∑(1 − (|actual heartbeats − reported heartbeats|)/actual heartbeats)(1)

For each participant, the mean of IAc in the three trials was calculated as a representative value for each condition and used for the statistical analyses. The IAc scores varied between 0 and 1, with higher scores indicating better heartbeat detection accuracy, reflecting a smaller difference between perceived and actual heartbeats. The IAc gain was computed as IAc in anticipation minus IAc in the baseline condition, with a higher score indicating higher IAc enhancement from baseline to anticipation condition.

SPSS 21.0 (IBM, New York, NY, USA) was employed to conduct the analysis. An independent sample *t*-test was utilized for group comparisons. Effects of the experimental manipulation on the dependent variables were compared using repeated-measures analyses of variance (ANOVA) and analysis of covariance (ANCOVA).

## 3. Results

Means and standard deviations of self-reported anxiety, heart rate, and, interoceptive accuracies among shy and non-shy adolescents in baseline and speech anticipation conditions are presented in Table 2.

### 3.1. Check on Manipulation

The effect of anticipation manipulation on self-reported anxiety mood and heart rate were analyzed in a 2 × 2 repeated measures analysis of variance (ANOVA), with condition (baseline vs. anticipation) as the within-subject factor, and group (shy vs. non-shy) as the between-subject factor.

For anxiety mood, there was a main effect of group, *F*(1, 59) = 12.88, *p* < 0.01, *η*_p_^2^ = 0.179, and a main effect of condition, *F*(1, 59) = 27.26, *p* < 0.001, *η*_p_^2^ = 0.316, while no interaction effect was observed, *F*(1, 59) = 0.19, *p =* 0.67, *η*_p_^2^ = 0.003.

For heart rate, there was a main effect of group, *F*(1, 59) = 5.06, *p* < 0.05, *η*_p_^2^ = 0.079, and a main effect of condition, *F*(1, 59) = 49.6, *p* < 0.001, *η*_p_^2^ = 0.457, while no interaction effect was observed, *F*(1, 59) = 0.01, *p =* 0.99, *η*_p_^2^ < 0.001.

Results indicated that, in both groups, the heart rate and anxiety mood in the anticipation condition was higher than that in the baseline condition. Results also indicated that, compared to the non-shy group, the shy group exhibited higher heart rate and anxiety mood regardless of condition.

### 3.2. Interoceptive Accuracy: The State and Trait Variable

To investigate the individual differences between shy and non-shy adolescents and the effect of the anxiety-eliciting situation on IAc, the heartbeat perception accuracy was investigated in a 2 × 2 ANOVA with condition as the within-subject factor and group as the between-subject factor. There was a main effect of condition, *F*(1, 59) = 8.37, *p* < 0.01, *η*_p_^2^= 0.124, and a main effect of group, *F*(1, 59) = 18.51, *p* < 0.001, *η*_p_^2^ = 0.239, with no interaction effect between condition and group, *F*(1, 59) = 0.36, *p* = 0.55, *η*_p_^2^ = 0.006.

To control the effect of trait anxiety on heartbeat perception accuracy, a 2 × 2 analysis of covariance (ANCOVA) with condition as the within-subject factor, group as the between-subject factor, and trait anxiety as a covariate was conducted. There was a main effect of group, *F*(1, 58)=11.06, *p <* 0.01, *η*_p_^2^= 0.160. There was no main effect of condition, *F*(1, 58) = 0.34, *p =* 0.56, *η*_p_^2^= 0.006, nor interaction effect between condition and group, *F*(1, 58) = 0.13, *p =* 0.73, *η*_p_^2^ = 0.002.

The IAc of participants in both the shy and non-shy groups increased in anticipation condition. Meanwhile, the shy group had higher IAc compared to the non-shy group regardless of experimental condition. This difference was still significant after controlling for the effect of trait anxiety. As for the IAc gain of shy and non-shy groups, the independent sample *t*-test revealed no significant difference, *t*(59) = 0.60, *p* = 0.55.

## 4. Discussion

The current study investigated differences between shy and non-shy individuals in interoceptive accuracy at baseline and during anticipation of a speech in Chinese adolescents. The two-way factorial design results supported both Hypothesis 1 and Hypothesis 2. Shy adolescents were consistently better at detecting their heartbeat than non-shy adolescents at baseline and during speech anticipation. Meanwhile, both groups’ participants were significantly more accurate in interoception in speech anticipation condition than in baseline condition. This result supports the prediction that a state anxiety manipulation would bring about heightened IAc. However, the difference in IAc gain between the shy group and the non-shy group was not significant, indicating that Hypothesis 3 was not supported in this study. This section will discuss these findings.

### 4.1. Speech Anticipation Enhances IAc

In both groups, IAc in anticipation was higher than that in the baseline condition. Prior studies have revealed IAc’s fluctuation due to physical activity [45] or in situations of heightened self-focus [22]. Our results are in line with the study of Durlik et al., which suggested that anxiety-eliciting situations (i.e., speech anticipation) can enhance IAc [23]. The current study verifies IAc’s state feature in a younger population of adolescents with high and low shyness levels. This evidence further suggests the interoceptive ability one possesses may change in response to the organism’s state. Both the heart rate and the anxiety mood in anticipation were significantly higher than that in the baseline, which confirmed that our manipulation successfully induced anxiety. Shyness typically occurs in social novelty and social interaction situations, which are anxiety-eliciting stimuli. The enhancement of IAc by this situational stimulus may account for more significant shyness features in such situations, especially physiological (e.g., racing heart) and emotional (e.g., social anxiety, fear) features.

### 4.2. Shy Adolescents Have Better Heartbeat Perception

This study found that shy adolescents were consistently more sensitive to interoceptive cues. This observed heightened interoceptive accuracy in shy adolescents confirmed the trait feature of IAc among the shy population. Prior studies have demonstrated the individual differences among social anxiety [19]. Consequently, interoceptive cues are posited to be more readily perceived, which may lead to their misinterpretation as symptoms of anxiety and arousal, visible to external observers, consequently bringing about an increase in anxiety [46]. The current study, to our knowledge, was the first study to investigate the individual difference of IAc in the shy adolescent population.

It should be noted that there are both adaptive and maladaptive views on IAc. On the one hand, some studies indicated that cardiac IAc is positively related to various physical and mental health indicators, which support the adaptive view on IAc. The beneficial functions of cardiac IAc, such as emotion perception/regulation, empathy, or self-regulation, were found [47,48,49], and lower cardiac IAc has been found in individuals with a range of psychopathologies, including anorexia nervosa and autism [12,50]. On the other hand, there is also a maladaptive view based on studies showing enhanced cardiac IAc in people with anxiety or panic disorders, negatively affecting their well-being [51,52,53]. While there is a wealth of evidence for both views, one study explored the mediating role of personality in the relationship between IAc and subjective well-being and found that, for optimistic participants, IAc did not predict higher well-being; for pessimistic individuals, it predicted lower well-being [54]. Herbert and Pollatos argued that feeling one’s heartbeat can both be read as feedback that the organism is functioning well and indicate that something is wrong with the organism [55]. While shy adolescents might feel their heartbeat more frequently than non-shy, it does not follow that they will interpret it more positively or negatively. Thus, future studies on the differences between shy and non-shy individuals in interpreting interoceptive signals should be a promising direction for revealing the mechanism of shyness.

The IAc gain resulting from anticipation in the shy group was not significantly different from that in the non-shy group. As shy individuals are theoretically higher in fear of negative evaluation than non-shy individuals, this result seems inconstant with the existing studies on the normal population, which revealed that higher fear of negative evaluation was related to a larger increase in IAc anticipatory anxiety [23]. This discrepancy might be due to the sample differences between Durlik et al. and this study. This study recruited adolescents with extreme high and low shyness levels as participants. As indicated by our results, shy participants in this study possessed higher IAc than non-shy participants in baseline conditions. In this regard, the shy adolescents might face a ‘ceiling effect’ regarding IAc enhancement. The study of Ainley et al. also suggested this possible effect, in which an increase in IAc during mirror self-observation was found in people with low IAc [22].

### 4.3. Limitation and Implications

This study has three major limitations. First, though mental tracking task is probably the most widely-used measure of IAc in interoception research [18], there is still an ongoing debate about its validity as an interoception measurement [56], as well as the validity of its scores [57,58]. Thus, it is important to replicate our research, and the protocol of Ferentzi et al. may be used as a multi-dimensional measurement of IAc [59]. Second, this study has only compared the Chinese adolescents with extreme high and low shyness, leaving those ordinary adolescents unexplored. In the future, studies on the effect of anxiety eliciting situation on IAc in adolescents with the mean level of shyness should be conducted. Third, the age range of the sample could be expanded. This study only focused on junior secondary students (ages 12–14), leaving high school students untouched. Future studies should cover the whole age range of secondary schools (ages 12–18), the time of ‘storm and stress’ and the critical period of psychological development.

Nevertheless, the findings of this study have important practical implications. First, attentional retraining could be a promising method for reducing exposure to interoceptive cues. Shy adolescents should practice diverting attention away from internal physical symptoms to the actual task. It has been proven to be effective in those with social anxiety [60], and is a key component of SP’s current cognitive treatments [61]. Second, cognitive restructuring methods could buffer the possible dysfunctional appraisal of physical symptoms that shy individuals may possess. Prior study with SP participants indicated that individuals with SP are prone to judge interoceptive cues as visible, which supposedly leads to a negative evaluation [46]. Accordingly, cognitive restructuring of dysfunctional beliefs about physical symptoms and their visibility may dampen the enhanced interoception among shy individuals.

## 5. Conclusions

To our knowledge, this study is the first empirical study on IAc differences between shy and non-shy adolescents. Our results provide additional cross-cultural evidence to support the state-trait model of IAc proposed in the study of Durlik et al., indicating that IAc could be regarded as both a state and trait variable [23]. This evidence about Chinese adolescents indicates that shy adolescents possess higher interoceptive accuracy, and the anxiety-eliciting situation could enhance adolescents’ IAc. These findings also shed light on our understanding of the mechanism of shyness and interventions.

## Figures and Tables

**Table 1:** Demographic characteristics of the sample: Means (with standard deviations), and *t*-test statistics for group comparisons.

	Non-Shy (*n* = 31)	Shy (*n* = 30)	*t*
Girl	16	16	
Boy	15	14	
Age	13.16 (0.86)	13.03 (0.67)	0.65
Body mass index	18.98 (3.32)	20.10 (2.59)	−1.48
Shyness-screening	20.75 (1.52)	46.33 (2.28)	−51.72 ***
Trait anxiety	37.81 (6.37)	49.32 (7.17)	−6.64 ***
Shyness-retest	26.97 (6.09)	43.30 (6.57)	−10.08 ***

Note. ***, *p* < 0.001; all df s of the t-test were 59.

**Table 2:** Means and standard deviations of self-reported anxiety, heart rate, and interoceptive accuracies (IAc) for shy and non-shy adolescents in baseline and speech anticipation conditions.

	Baseline			Anticipation			IAc Gain
	Anxiety Mood (VAS)	Heart Rate (bpm)	IAc	Anxiety Mood (VAS)	Heart Rate (bpm)	IAc	
Non-shy *n* = 31	11.90 (3.46)	86.03(12.33)	0.29 (0.26)	14.87 (4.64)	92.98 (12.97)	0.37 (0.29)	0.08 (0.19)
Shy *n* = 30	15.97 (5.53)	92.68(12.10)	0.55 (0.16)	19.47 (7.00)	99.62 (11.14)	0.60 (0.21)	0.05 (0.17)

Note. IAc = Interoceptive accuracy.

**Figure A1:**
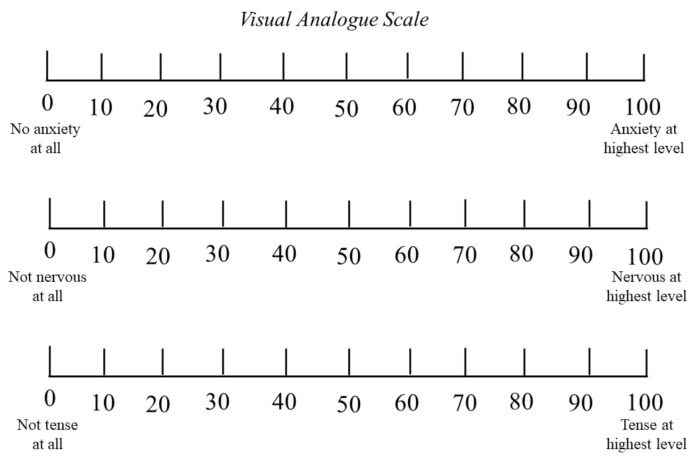
The visual analogue scale for anxiety mood.

## Data Availability

The data presented in this study are available on request from the corresponding author.

## Appendix A

Chinese version of Cheek and Buss shyness scale.

INSTRUCTIONS: Please read each item carefully and decide to what extent it is characteristic of your feelings and behavior. Fill in the blank next to each item by choosing a number from the scale printed below.

Response options:

1 = Strongly disagree, 2 = Agree, 3 = Neutral, 4 = Disagree, 5 = Strongly agree.

Items:

____ 1. I feel tense when I’m with people I don’t know well.

____ 2. I am somewhat socially awkward.

____ 3. I do not find it difficult to ask other people for information.

____ 4. I am often uncomfortable at parties and other social functions.

____ 5. When in a group of people, I have trouble thinking of the right things to talk about.

____ 6. It does not take me long to overcome my shyness in new situations.

____ 7. It is hard for me to act naturally when I am meeting new people.

____ 8. I feel nervous when speaking to someone in authority.

____ 9. I have no doubts about my social competence.

____ 10. I have trouble looking someone right in the eye.

____ 11. I feel inhibited in social situations.

____ 12. I do not find it hard to talk to strangers.

____ 13. I am shyer with members of the opposite sex.

Note. Items 3, 6, 9, and 12 are reversed and recoded before scoring: (1 = 5) (2 = 4) (4 = 2) (5 = 1). The score is the sum of all item scores.

## Appendix B

The trait subscale of the Chinese Version State-Trait-Anxiety-Inventory.

INSTRUCTIONS: Several statements that people use to describe themselves are given below. Read each statement and then circle the appropriate number to the right of the statement to indicate how you feel generally.

Response options:

Almost never (1); Sometimes (2); Often (3); Almost always (4).

Items:

21. I feel pleasant. (*)

22. I feel tension or turmoil.

23. I feel self-satisfaction. (*)

24. I wish I could be as happy as others. (*)

25. I feel strained.

26. I feel rested. (*)

27. I am calm, cool, and collected. (*)

28. I feel difficulties pile up and become hard to overcome.

29. I worry too much about things that actually do not matter.

30. I am happy. (*)

31. My mind is in a state of chaos.

32. I feel a lack of self-confidence.

33. I feel secure. (*)

34. I can easily make up my mind. (*)

35. I feel blue.

36. I feel content. (*)

37. I feel that unimportant thoughts surround me and bother me.

38. I feel very upset and take disappointment badly.

39. I am a steady person. (*)

40. I feel nervous when I think about my current situation and interests.

Note: Items with (*) are reversed and recoded before scoring: (1 = 4) (2 = 3) (3 = 2) (4 = 1). The score is the sum of all item scores.

## Appendix C

The anxiety mood of participants was measured with a visual analogue scale (see below). Participants were asked to report a number between 0 and 100 to indicate their anxiety mood.
